# Loureirin B Reduces Insulin Resistance and Chronic Inflammation in a Rat Model of Polycystic Ovary Syndrome by Upregulating GPR120 and Activating the LKB1/AMPK Signaling Pathway

**DOI:** 10.3390/ijms252011146

**Published:** 2024-10-17

**Authors:** Jing Wang, Zheng Huang, Zhiyong Cao, Yehao Luo, Yueting Liu, Huilu Cao, Xiusong Tang, Gang Fang

**Affiliations:** 1 Guangxi Key Laboratory for Applied Fundamental Research of Zhuang Medicine-Key Laboratory Project under Guangxi Health Commission, Guangxi University of Chinese Medicine, Nanning 530001, Chinacaozhiyong2019@163.com (Z.C.); lxmtxs2024@163.com (X.T.); 2 Guangxi Higher Education Key Laboratory for the Research of Du-Related Diseases in Zhuang Medicine, Guangxi University of Chinese Medicine, Nanning 530001, China; 3 Health Science Center, Hubei Minzu University, Enshi 445000, China; 4 The Second Clinical College of Guangzhou University of Chinese Medicine, Guangzhou 510006, China; docluoyehao@163.com

**Keywords:** polycystic ovary syndrome, GPR120, loureirin B, insulin resistance, inflammation, LKB1/AMPK signaling pathway

## Abstract

Polycystic ovary yndrome (PCOS) is a common metabolic disorder in women, which is usually associated with insulin resistance (IR) and chronic inflammation. Loureirin B (LrB) can effectively improve insulin resistance and alleviate chronic inflammation, and in order to investigate the therapeutic effect of LrB on polycystic ovary syndrome with insulin resistance (PCOS-IR), we conducted animal experiments. A PCOS-IR rat model was established by feeding a high-fat diet combined with letrozole (1 mg/kg·d for 21 days). The rats were treated with the GPR120 agonists TUG-891 and LrB for 4 weeks. Biochemical parameters (fasting blood glucose, total cholesterol, triglycerides, high- and low-density lipoprotein), hormone levels (serum insulin, E2, T, LH, and FSH), and inflammatory cytokines (TNF-α, IL-1β, IL-6, and IL-18) were analyzed. Histopathological analyses of ovaries were performed using hematoxylin/eosin (H&E) staining. Real-time PCR and western blotting were used to assess GPR120, NLRP3, and caspase-1 expression in ovaries, and immunohistochemistry was used to evaluate LKB1 and AMPK protein expression. LrB reduced body weight, Lee’s index, ovarian index, ovarian area, and volume in PCOS-IR rats. It lowered fasting blood glucose, serum insulin, and HOMA-IR. LrB decreased total serum cholesterol, triglyceride, and LDL levels and increased HDL levels. It reduced serum T, LH, and LH/FSH and raised serum E2 and FSH levels. LrB downregulated the mRNA and protein expression levels of NLRP3 and Caspase-1, increased the protein and mRNA expression levels of GPR120 in rat ovaries, and increased LKB1 and AMPK protein expression in ovaries, ameliorating ovarian histopathological changes in PCOS-IR rats. Taken together, LrB upregulated GPR120, LKB1, and AMPK protein expression, downregulated NLRP3 and Caspase-1 protein expression, reduced insulin resistance and chronic inflammation, and ameliorated histopathological changes in ovarian tissues in PCOS rats, suggesting its potential as a treatment for PCOS.

## 1. Introduction

Polycystic ovary syndrome (PCOS) is a common endocrine disorder in women of reproductive age, with an incidence of 6–20% [1]. Its symptoms include irregular menstrual cycles, dysfunctional ovulation, polycystic ovarian follicles, and hyperandrogenism [2]. Insulin resistance also often occurs in PCOS; it is present in up to 75% of patients [3] and is closely related to female reproductive function [4]. Excess insulin stimulates the ovarian stroma in women with PCOS to release testosterone [5], exacerbating the symptoms. Additionally, chronic inflammation is observed in PCOS patients [6], where sustained release of inflammatory mediators impairs glucose metabolism and worsens insulin resistance [7], posing serious health risks to reproductive-age women. Its incidence tends to be higher in younger women with PCOS who could develop a state of pre-diabetes or even diabetes before the age of 40. But at the current state of the art, there are no therapeutic tools available other than a hormonal treatment, a restrictive diet, or the proposal to use metformin with a discrete improvement in the hormonal balance useful for ovulation. Moreover, long-term use of metformin is often accompanied by gastrointestinal and other side effects [8,9]. Therefore, searching for effective natural medicines with minimal side effects is urgent.

G-protein-coupled receptor 120 (GPR120, free fatty-acid receptor 4, FFAR4) is highly expressed in adipose tissue and functions as a lipid sensor [10]. It is expressed in different tissues and cells and has a role in energy regulation, as an anti-inflammatory, and as a potent insulin sensitizer [11]. Studies have shown that GPR120 protects rats against the harmful effects of PCOS by lowering insulin resistance, reducing lipid accumulation and serum testosterone levels, and preventing histopathological changes in ovaries [12]. Moreover, GPR120 agonists inhibit activation of NOD-like receptor pyrin domain-containing three (NLRP3) inflammasomes, which prevents apoptosis of retinal endothelial cells from high glucose levels; high-glucose environments decrease GPR120 protein expression [13]. Therefore, we hypothesized that the activation of GPR120 could offer a potentially effective therapeutic approach for treating PCOS.

Loureirin B (LrB, 1-(4-hydroxyphenyl)-3-(2,4,6-trimethoxyphenyl)propan-1-one) is one of the active ingredients isolated from the traditional Chinese medicine formulation, Resina Draconis [14]. It has various pharmacological effects, including antibacterial, anti-inflammatory, increasing insulin sensitivity, and reducing insulin resistance [15,16]. Previous studies have shown that LrB prevents diet-induced obesity in mice by activating the ω3 PUFA-GPR120-UCP1 axis in adipose tissue, thereby reducing the associated insulin resistance and systemic inflammation [17]. In HepG2 cells, it attenuated insulin resistance by regulating gluconeogenesis [18] and inhibited the release of inflammatory cytokines [19]. LrB activated GPR120 and regulated glucose and lipid metabolism with anti-inflammatory effects; however, whether LrB alters the pathology of PCOS-IR by regulating GPR120 expression remains unclear.

Thus, we determined the effect of LrB on GPR120 expression in the ovaries of PCOS-IR model rats. Subsequently, we explored the effects of LrB on the NLRP3 inflammasome and liver kinase B1 (LKB1)/AMP-activated protein kinase (AMPK) signaling pathways. Through these experiments, we aimed to provide evidence that LrB can be used as a treatment for PCOS-IR.

## 2. Results

**Figure 1:**
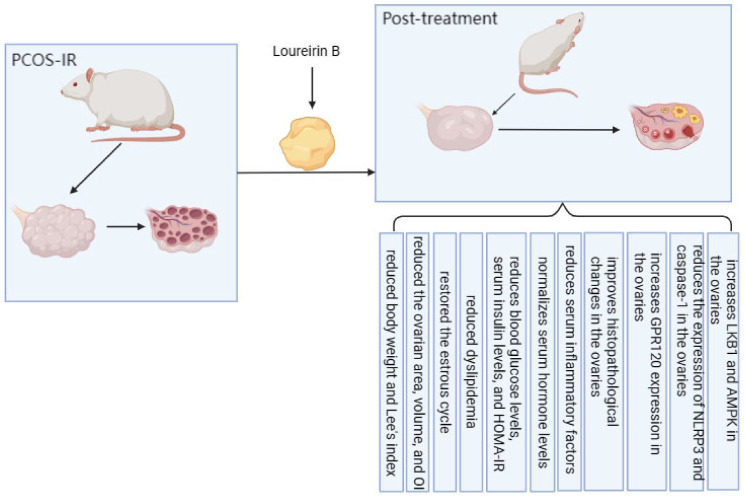
Therapeutic effect of LrB on PCOS-IR rats.

Figure 1 briefly summarizes the therapeutic effects of LrB in PCOS-IR rats.

### 2.1. LrB Reduced Body Weight and Lee’s Index in PCOS-IR Rats

**Figure 2:**
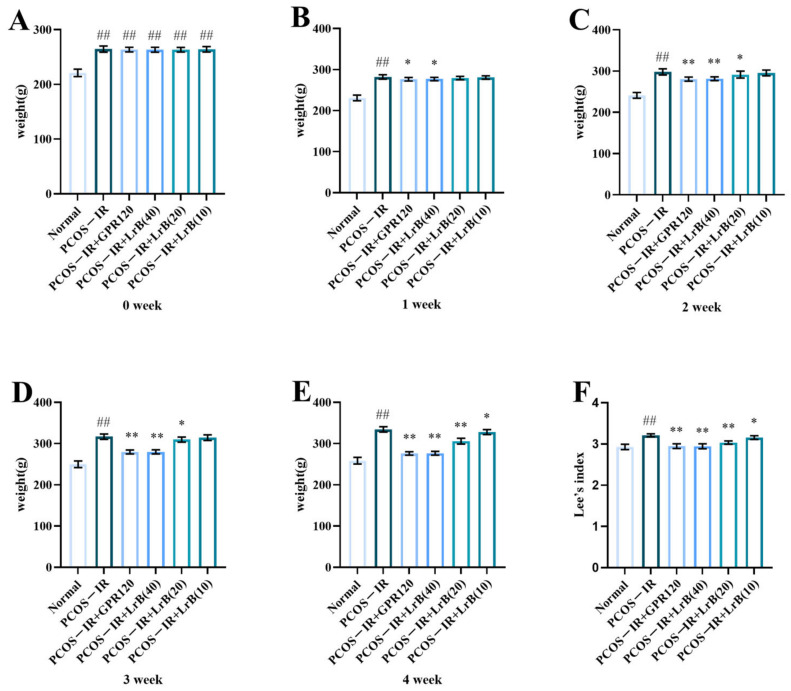
Effect of LrB on body weight and Lee’s index in PCOS-IR rats. The data are mean ± SDs, n = 10. Body weights of rats at (**A**) week 0 of treatment, (**B**) week 1, (**C**) week 2, (**D**) week 3, (**E**) week 4. (**F**) Lee’s indices of the rats in each group after four weeks of treatment. In comparison with the normal group, ^##^ *p <* 0.01; in comparison with the PCOS-IR group, * *p <* 0.05; ** *p <* 0.01.

In comparison with the normal group, the differences in body weights of the model rats were highly significant (Figure 2A). One week after administration, compared with the PCOS-IR group, both the PCOS-IR+GPR120 agonist group and the PCOS-IR+LrB (40 mg/kg) group showed significant reductions in body weight (Figure 2B). At weeks two and three of treatment, the PCOS-IR+GPR120 group and the PCOS-IR+LrB (40 mg/kg) group presented significant reductions in body weight (Figure 2C,D); the PCOS-IR+LrB (20 mg/kg) group also showed significant reductions in body weight (Figure 2C,D). By the fourth week of treatment, the PCOS-IR+GPR120 agonist group, the PCOS-IR+LrB (40 mg/kg) group, and the PCOS-IR+LrB (20 mg/kg) group all had significant reductions in body weight (Figure 2E); the PCOS-IR+LrB (10 mg/kg) group also showed significant decreases in body weight (Figure 2E).

After four weeks of treatment, the Lee index of the PCOS-IR group was significantly elevated (Figure 2F) in comparison with the normal group. Relative to the PCOS-IR group, the PCOS-IR+GPR120, PCOS-IR+LrB (40 mg/kg), and PCOS-IR+LrB (20 mg/kg) groups all presented significant decreases in Lee’s index (Figure 2F), as did the PCOS-IR+LrB (10 mg/kg) group (Figure 2F). The high dose of LrB (40 mg/kg) had effects on body weight and Lee’s index in PCOS-IR rats comparable to those of the GPR120 agonist.

### 2.2. LrB Reduced the Ovarian Area, Volume, and OI in PCOS-IR Rats

**Figure 3:**
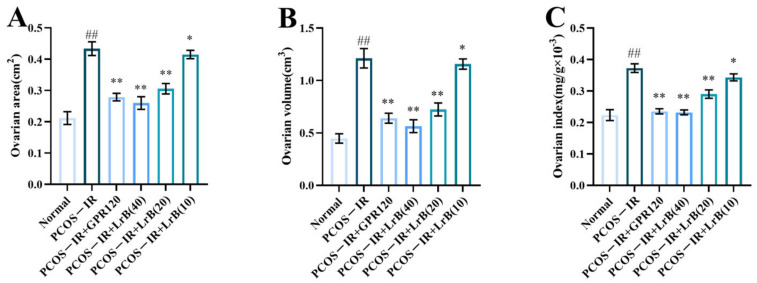
Effect of LrB on the ovarian area, ovarian volume, and ovarian index in PCOS-IR rats. The values are means ± SDs; n = 10. (**A**) Ovarian area at four weeks of treatment. (**B**) Ovarian volume at four weeks. (**C**) Ovarian indices at four weeks. In comparison with the normal group, ^##^ *p <* 0.01; in comparison with the PCOS-IR group, * *p <* 0.05; ** *p <* 0.01.

At 4 weeks of treatment, the ovarian area and volume and the ovarian index of the rats in the PCOS-IR group were significantly greater (Figure 3A,C) than those in the normal group. Compared with the PCOS-IR group, the area, volume, and index of the ovaries in the PCOS-IR+GPR120, PCOS-IR+LrB (40 mg/kg), and PCOS-IR+LrB (20 mg/kg) groups were significantly lower (Figure 3A,C). The area, volume, and OI in the PCOS-IR+LrB (10 mg/kg) group were also significantly decreased (Figure 3A,C). Additionally, high-dose LrB (40 mg/kg) had a therapeutic effect similar to that of the GPR120 agonist.

### 2.3. LrB Treatment Restored the Estrous Cycle in PCOS-IR Rats

**Figure 4:**
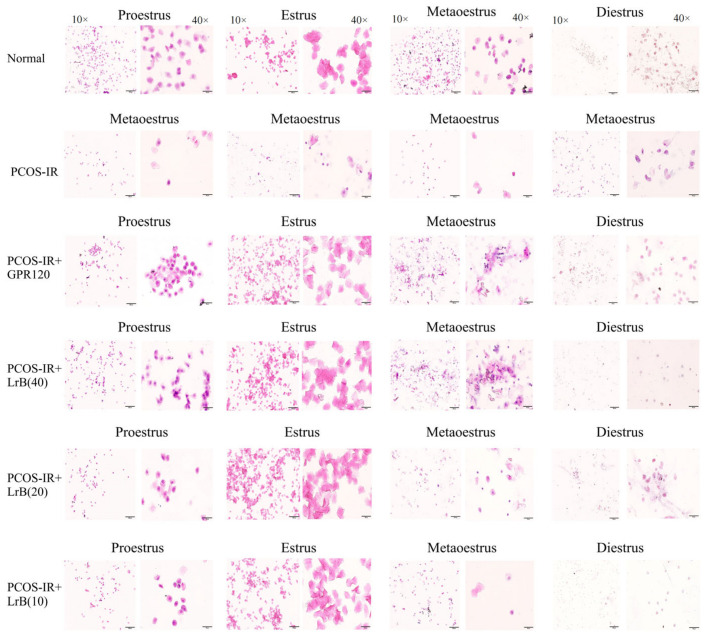
Vaginal cytology images (magnification 10×, 40×). LrB improved the estrous cycle in PCOS-IR rats.

Vaginal smear analysis revealed that at the beginning of this study, the rats exhibited normal estrous cycles. The proestrus phase is delineated by an abundance of nucleated epithelial cells, the estrus phase by cornified cells, and the metestrus phase by nucleated epithelial cells, cornified cells, and leukocytes, whereas the diestrus phase shows abundant leukocytes. After the induction of PCOS with letrozole, the rats exhibited disrupted estrous cycles, mostly manifesting in a prolonged diestrus phase. Treatment with the GPR120 agonist and LrB for four weeks improved the estrous cycles (Figure 4).

### 2.4. LrB Reduced Dyslipidemia in PCOS-IR Rats

**Figure 5:**
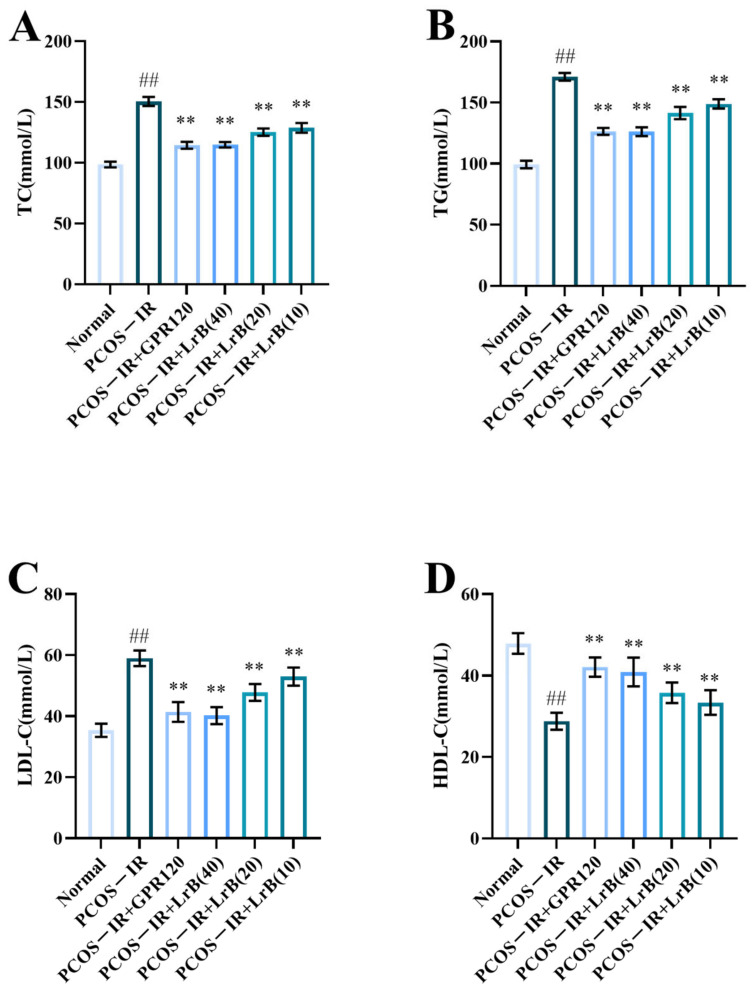
Effect of LrB on lipid metabolic activity in PCOS-IR rats. The values are means ± SDs; n = 10. (**A**) TC levels at four weeks of treatment. (**B**) TG levels at four weeks. (**C**) LDL levels at four weeks. (**D**) HDL levels at four weeks. Relative to the normal group, ^##^ *p <* 0.01; relative to the PCOS-IR group, ** *p <* 0.01.

The PCOS-IR group showed a significant increase in TC, TG, and LDL (Figure 5A,C) and a significant decrease in HDL (Figure 5D) in comparison with the normal group. The PCOS-IR+GPR120, PCOS-IR+LrB (40 mg/kg), PCOS-IR+LrB (20 mg/kg), and PCOS-IR+LrB (10 mg/kg) groups had significantly reduced levels of TC, TG, and LDL (Figure 5A,C) and a significantly increased HDL level (Figure 5D) in comparison with the PCOS-IR group. A high dose of LrB (40 mg/kg) affected TC, TG, LDL, and HDL levels comparable to those of the GPR120 agonist.

### 2.5. LrB Treatment Reduces Blood Glucose, Serum Insulin, and HOMA-IR in PCOS-IR Rats

**Figure 6:**
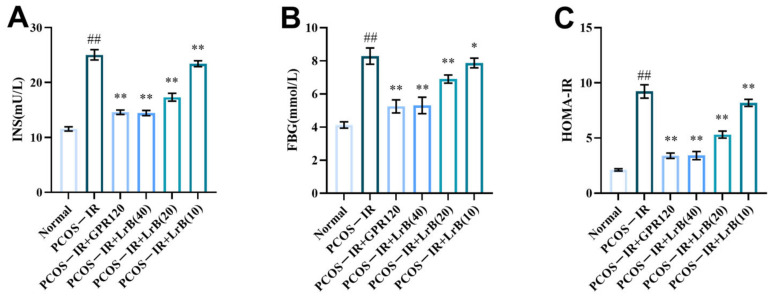
Effect of LrB on fasting blood glucose and serum insulin in PCOS-IR rats; values are presented as the means ± SDs, n = 10. (**A**) INS levels at four weeks of treatment. (**B**) FBG levels at four weeks. (**C**) HOMA-IR at four weeks. Relative to the normal group, ^##^ *p <* 0.01; relative to the PCOS-IR group, * *p <* 0.05; ** *p <* 0.01.

Insulin resistance is common in PCOS. To assess whether LrB affected insulin resistance in the PCOS-IR rat model, we measured FBG and INS concentrations. After four weeks of treatment, the PCOS-IR group had a significant increase in FBG and INS (Figure 6A,B) and markedly increased HOMA-IR (Figure 6C) in comparison with the normal group. In comparison with the PCOS-IR group, INS and FBG, as well as HOMA-IR, were significantly lower in the PCOS-IR+GPR120, PCOS-IR+LrB (40 mg/kg), and PCOS-IR+LrB (20 mg/kg) groups (Figure 6A,C). The PCOS-IR+LrB (10 mg/kg) group also showed significant decreases in INS and HOMA-IR (Figure 6A,C), as well as notable decreases in FBG levels (Figure 6B). Moreover, the effects of the high dose of LrB (40 mg/kg) were similar to the effects of the GPR120 agonist.

### 2.6. LrB Treatment Normalizes Hormone Concentration in Serum of PCOS-IR Rats

**Figure 7:**
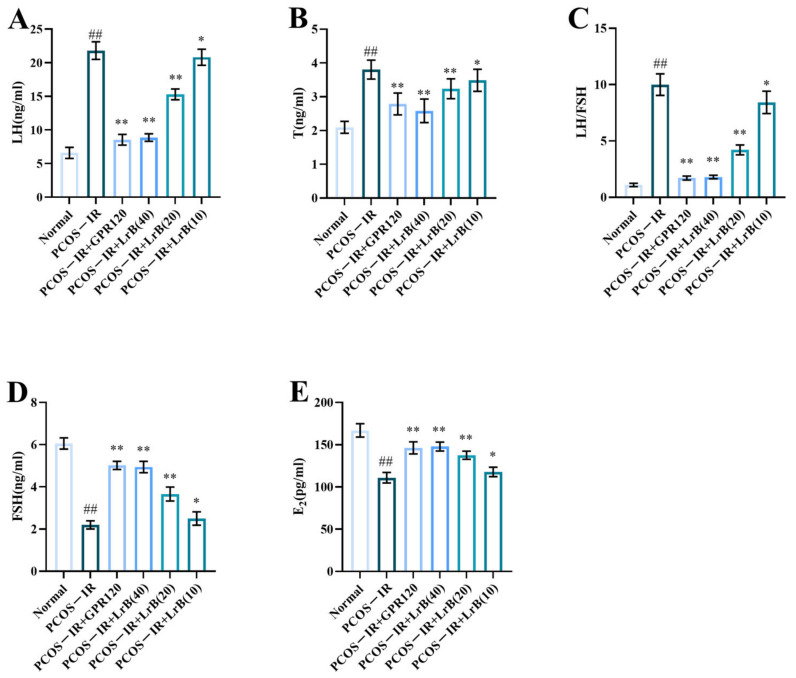
Effects of LrB on hormone levels in PCOS-IR rats; values are means ± SDs; n = 10. (**A**) LH levels at four weeks of treatment. (**B**) T levels at four weeks. (**C**) LH/FSH ratio at four weeks. (**D**) FSH levels at four weeks. (**E**) E2 levels at four weeks. Relative to the normal group, ^##^ *p <* 0.01; relative to the PCOS-IR group, * *p <* 0.05; ** *p <* 0.01.

Sex hormone imbalance and anovulation or oligo-ovulation are important clinical features of PCOS. After four weeks of LrB treatment, the PCOS-IR group animals presented significant increases in serum LH and T and LH/FSH ratio (Figure 7A,C) and significant decreases in FSH and E2 (Figure 7D,E) in comparison with the normal group. The PCOS-IR+GPR120 agonist, PCOS-IR+LrB (40 mg/kg), and PCOS-IR+LrB (20 mg/kg) groups presented significant decreases in LH and T and LH/FSH ratio (Figure 7A,C) and significant increases in FSH and E2 levels (Figure 7D,E) in comparison with PCOS-IR. The PCOS-IR+LrB (10 mg/kg) group presented significant decreases in LH and T and LH/FSH ratio (Figure 7A,C) and significant increases in FSH and E2 (Figure 7D,E). Moreover, the therapeutic effect of LrB (40 mg/kg) was like that of the GPR120 agonist.

### 2.7. LrB Treatment Reduces Serum TNF-α, IL-1β, -6, and -18 in PCOS-IR Rats

**Figure 8:**
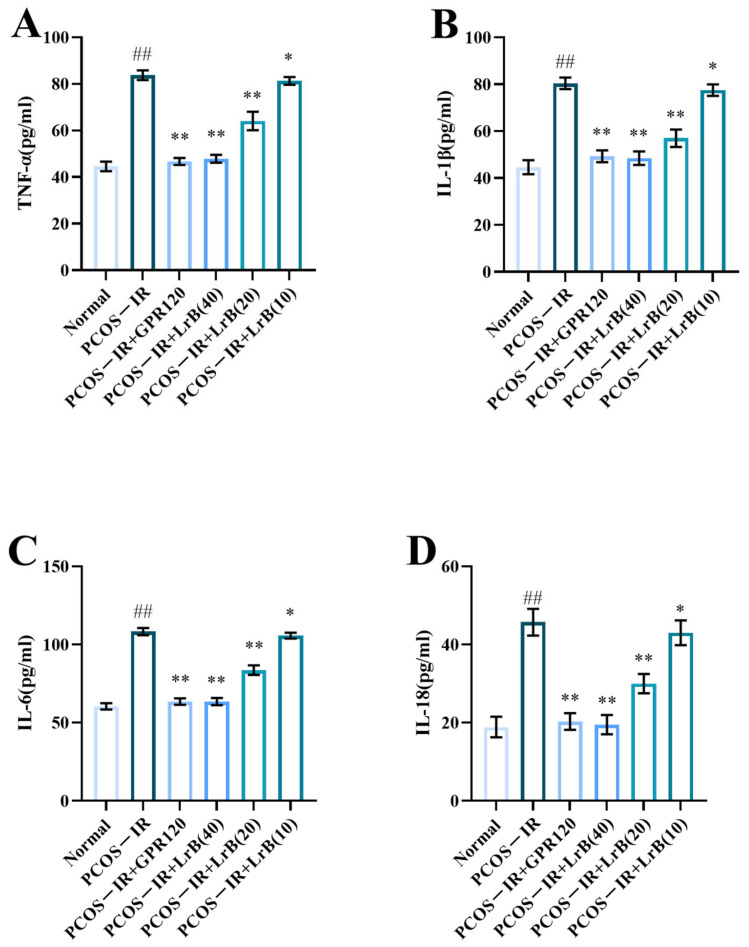
LrB alleviates the serum inflammatory response in PCOS-IR rats. Values are means ± SDs; n = 10. (**A**) Serum TNF-α content at four weeks of treatment. (**B**) Serum IL-1β at four weeks. (**C**) Serum IL-6 at four weeks. (**D**) Serum IL-18 at four weeks. Relative to the normal group, ^##^ *p <* 0.01; relative to the PCOS-IR group, * *p <* 0.05, ** *p <* 0.01.

Serum content of TNF-α, IL-1β, -6, and -18 showed a significant elevation in the PCOS-IR group after four weeks of treatment (Figure 8A,D) in comparison with normal rats. The PCOS-IR+GPR120, PCOS-IR+LrB (40 mg/kg), and PCOS-IR+LrB (20 mg/kg) groups had a significantly lower content of TNF-α, IL-1β, IL-6, and IL-18 (Figure 8A,D), whereas the PCOS-IR+LrB (10 mg/kg) group presented significantly lower levels of these factors (Figure 8A,D) in comparison with the PCOS-IR group. The therapeutic effect of high-dose LrB (40 mg/kg) was like that of the GPR120 agonist.

### 2.8. LrB Improves Histopathological Changes in PCOS-IR Rat Ovaries

**Figure 9:**
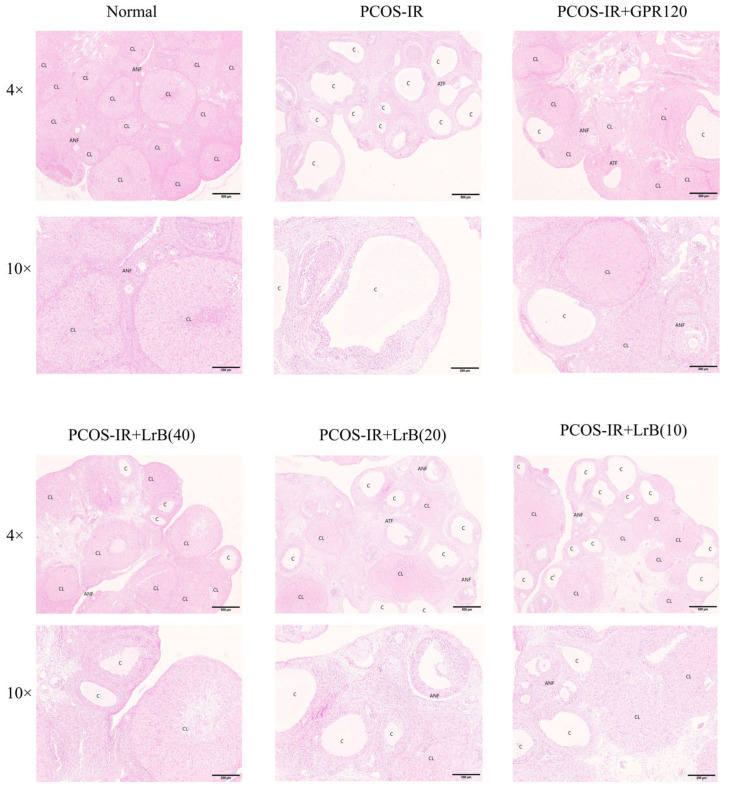
Effects of LrB treatment on morphological changes in ovarian tissue (magnification 4×, 10×). Representative H&E-stained ovarian tissue sections after four weeks of treatment. C, cystic follicles; CL, corpus luteum; ANF, antral follicles; ATF, atretic follicles.

At 4 weeks of treatment, the PCOS-IR group presented cystic ovarian follicles, a reduced granulosa cell layer, a loss of oocytes, and fewer corpora lutea. The GPR120 agonist, PCOS-IR+LrB (40 mg/kg), PCOS-IR+LrB (20 mg/kg), and PCOS-IR+LrB (10 mg/kg) groups presented significantly fewer cystic follicles, more granulosa cell layers, more oocytes, and more corpora lutea (Figure 9) relative to the PCOS-IR group. The therapeutic effect of LrB (40 mg/kg) was like that of the GPR120 agonist.

### 2.9. LrB Increases GPR120 Expression in PCOS-IR Rat Ovaries

**Figure 10:**
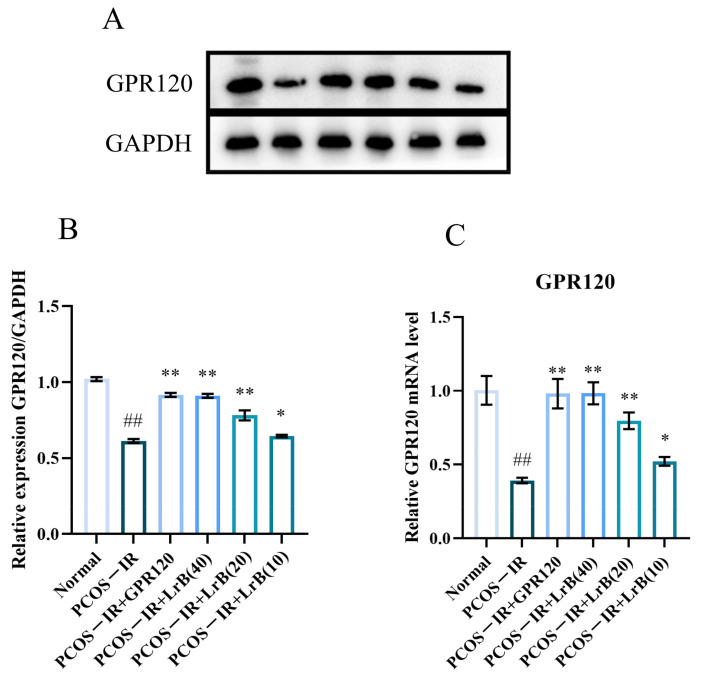
Effects of LrB on GPR120 expression in PCOS-IR rat ovaries. Data are means ± SDs; n = 3. (**A**) Western blot analysis of GPR120 and GAPDH in ovarian tissues at four weeks of treatment. (**B**) Quantitative analysis of GPR120 levels in ovarian tissues at four weeks of treatment. (**C**) Expression levels of GPR120 mRNA in ovarian tissues at four weeks of treatment. Relative to the normal group, ^##^ *p <* 0.01; relative to the PCOS-IR group, * *p <* 0.05; ** *p <* 0.01.

GPR120 mediates chronic inflammation and insulin resistance and has a crucial part in PCOS. Therefore, we evaluated the expression levels of GPR120 in model animals by RT-qPCR and WB. In comparison with the normal group, both the transcription and the translation of GPR120 were significantly lower in the PCOS-IR rats at 4 weeks of treatment (Figure 10A,C). In comparison with the PCOS-IR group, the PCOS-IR+GPR120 agonist, PCOS-IR+LrB (40 mg/kg), and PCOS-IR+LrB (20 mg/kg) groups presented significant increases in GPR120 mRNA and protein (Figure 10A,C), while the PCOS-IR+LrB (10 mg/kg) group showed significant increases in GPR120 mRNA and protein expression (Figure 10A,C). Additionally, the therapeutic effect of LrB (40 mg/kg) was comparable to the GPR120 agonist.

### 2.10. LrB Reduces the Expression of NLRP3 and Caspase-1 in the Ovaries of PCOS-IR Rats

**Figure 11:**
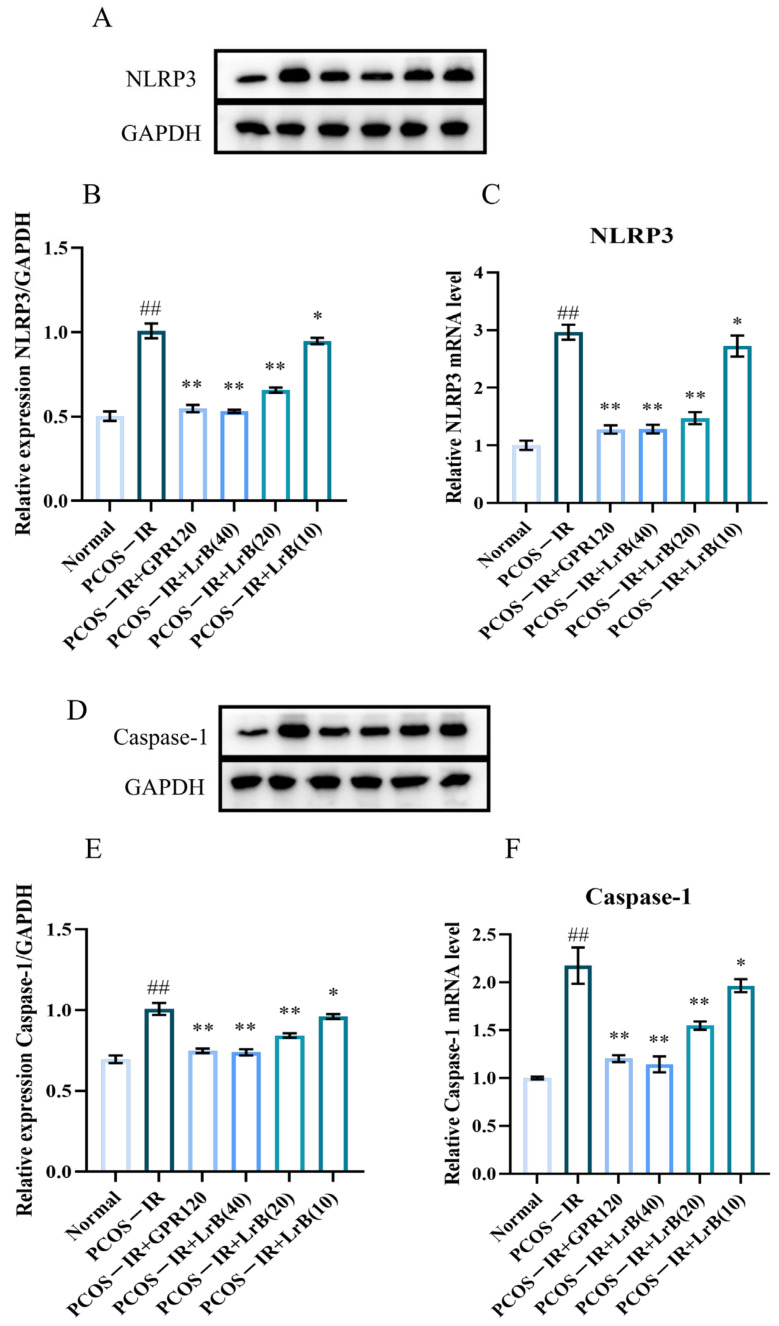
Effects of LrB on NLRP3 and Caspase-1 levels in the ovaries of PCOS-IR rats. The values are means ± SDs; n = 3. (**A**) Western blot analysis of NLRP3 and GAPDH in ovarian tissues at four weeks of treatment. (**B**) Quantitative analysis of NLRP3 levels in ovarian tissues at four weeks of treatment. (**C**) Expression levels of NLRP3 mRNA in ovarian tissues at four weeks of treatment. (**D**) Western blot analysis of Caspase-1 and GAPDH in ovarian tissues at four weeks of treatment. (**E**) Quantitative analysis of Caspase-1 levels in ovarian tissues at four weeks of treatment. (**F**) Expression levels of Caspase-1 mRNA in ovarian tissues at four weeks of treatment. Relative to the normal group, ^##^ *p <* 0.01; relative to the PCOS-IR group, * *p <* 0.05; ** *p <* 0.01.

The expression of NLRP3 inflammasomes and Caspase-1 in the ovaries of different groups of animals was determined by real-time RT—qPCR and western immunoblotting. In comparison with the normal group, both mRNA and protein of NLRP3 and Caspase-1 were significantly increased in the PCOS-IR group after four weeks of treatment (Figure 11A,F). In comparison with the PCOS-IR group, the PCOS-IR+GPR120 agonist, PCOS-IR+LrB (40 mg/kg), and PCOS-IR+LrB (20 mg/kg) groups presented significant decreases in NLRP3 and Caspase-1 (Figure 11A,F), while the PCOS-IR+LrB (10 mg/kg) group presented significant decreases in NLRP3 and caspase-1 (Figure 11A,F). The therapeutic effect of LrB (40 mg/kg) was like that of the GPR120 agonist.

In summary, these data demonstrate the significant anti-inflammatory properties of the GPR120 agonist and LrB in letrozole-induced PCOS-IR.

### 2.11. LrB Increases LKB1 and AMPK in PCOS-IR Rat Ovaries

**Figure 12:**
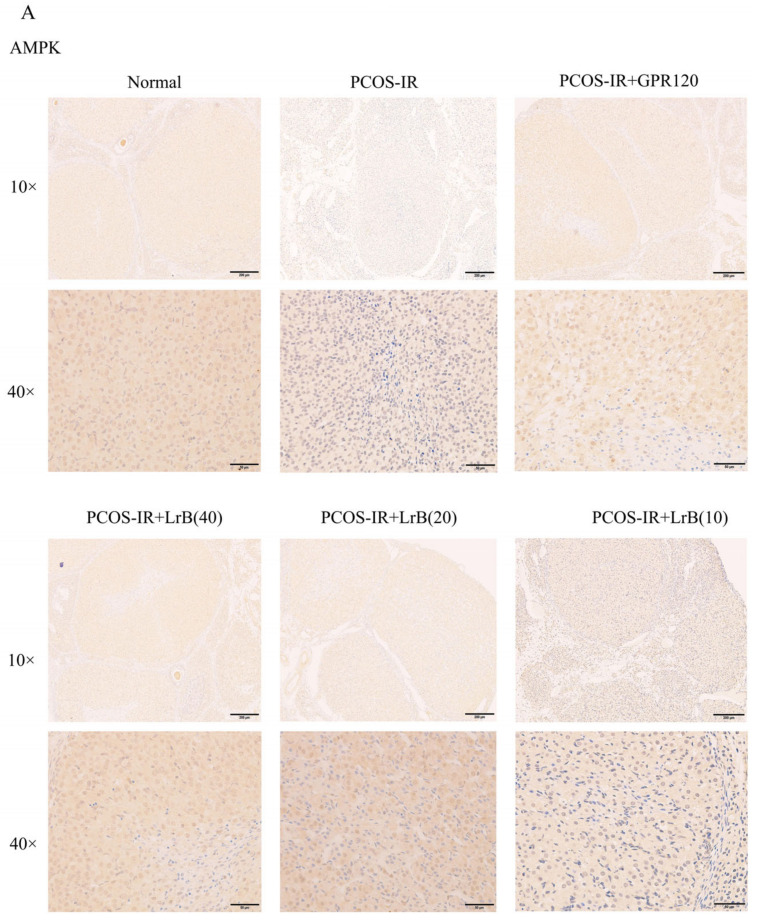

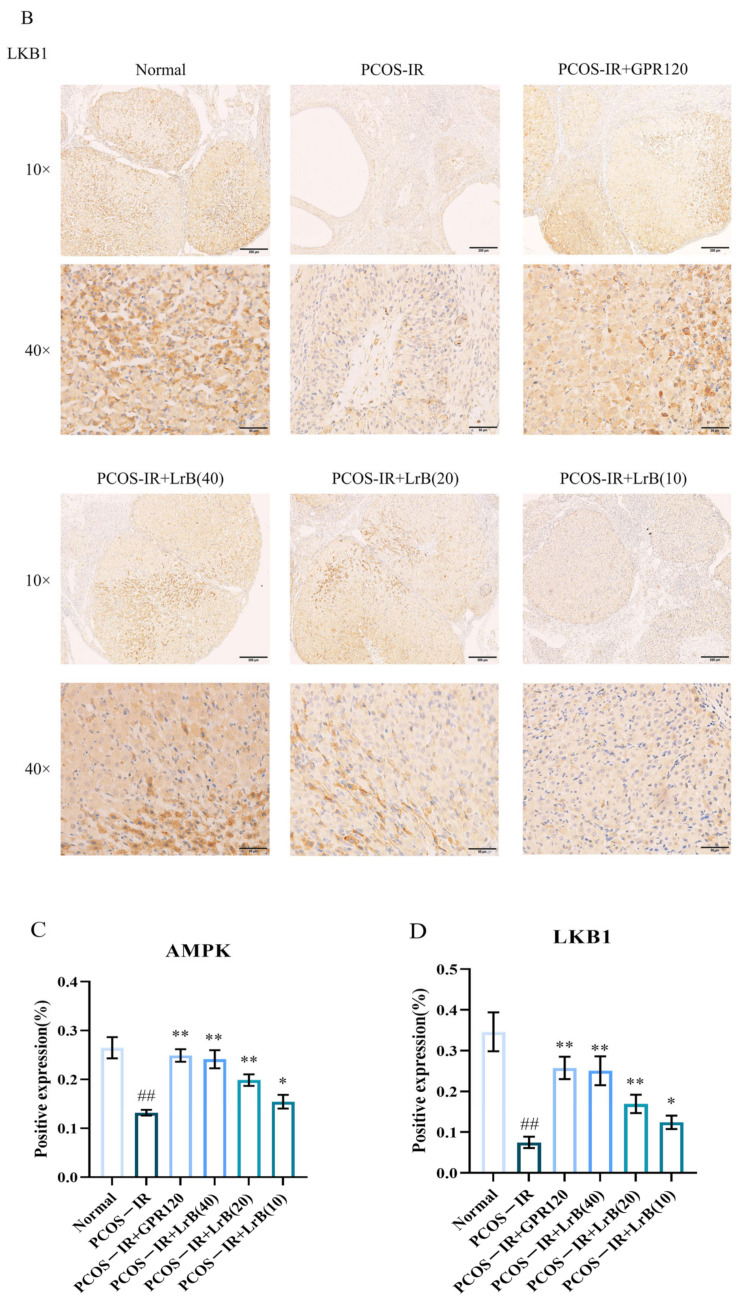
The influence of LrB on expression of LKB1 and AMPK in the ovaries of PCOS-IR rats. The values are means ± SDs; n = 5. (**A**) Expression of AMPK in the ovarian tissues of each group at four weeks of treatment (magnification 10×, 40×). (**B**) Expression of LKB1 in the ovarian tissues of each group at four weeks of treatment (magnification 10×, 40×). (**C**) Positive expression of AMPK was analyzed. (**D**) Positive expression of LKB1 was analyzed. Relative to the normal group, ^##^ *p <* 0.01; relative to the PCOS-IR group, * *p <* 0.05; ** *p <* 0.01.

We employed immunohistochemistry (IHC) to assess the expression of LKB1 and AMPK in rat ovaries. In comparison with the normal group, the expression of LKB1 and AMPK in the ovarian tissues of PCOS-IR rats showed a significant reduction after four weeks of treatment (Figure 12A,D). Compared with the PCOS-IR group, the PCOS-IR+GPR120 agonist, PCOS-IR+LrB (40 mg/kg), and PCOS-IR+LrB (20 mg/kg) groups presented significant increases in the expression levels of LKB1 and AMPK in ovarian tissues (Figure 12A,D), whereas the PCOS-IR+LrB (10 mg/kg) group presented significant increases in the expression of LKB1 and AMPK (Figure 12A,D). These results indicate that LrB increases LKB1 and AMPK in PCOS-IR rat ovaries, and the therapeutic effect of LrB (40 mg/kg) is similar to that of the GPR120 agonist.

## 3. Discussion

LrB has been shown to be beneficial in treating insulin resistance [18] and immune suppression [20], but its therapeutic effects and underlying mechanisms in individuals with PCOS are still unclear. Here, we showed that LrB can upregulate GPR120, LKB1, and AMPK in PCOS-IR rat ovaries, downregulate NLRP3 and Caspase-1, reduce insulin resistance, alleviate chronic inflammation, and ameliorate symptoms in PCOS-IR rats, by restoring the estrous cycle, reducing the number of cystic follicles, and normalizing hormone levels.

Insulin resistance is central to the pathogenesis of PCOS, where prolonged IR can lead to hyperinsulinemia, exacerbating PCOS symptoms [21,22] and resulting in anovulation and poor follicular development. After LrB treatment, FBG and serum INS levels in PCOS-IR rats were significantly lower than in the control. IR also contributes to dyslipidemia in PCOS patients, leading to elevated total serum cholesterol, triglycerides, and LDL and decreased HDL [23]. Following LrB treatment, however, FBG and INS levels in PCOS-IR rats significantly decreased, along with a significant lowering of total serum cholesterol, triglycerides, and LDL and a significant increase in HDL.

GPR120 is a free fatty acid receptor that regulates lipid and glucose metabolism and improves inflammatory diseases [24]. Studies have shown that GPR120 agonists could increase GPR120 protein expression in PCOS-IR rat ovaries, improving insulin sensitivity and ovarian function [12]. The LKB1/AMPK pathway is associated with the onset of IR [25]. When the ratio of AMP to ATP increases, AMP binds to the AMPK-γ regulatory subunit and phosphorylates LKB1. P-LKB1 then binds to the Thr172 site on the AMPK-α subunit, phosphorylating it to P-AMPK and initiating metabolic pathways to increase ATP levels while shutting down synthetic pathways to reduce ATP consumption and maintain energy balance [26]. Therefore, PCOS-associated IR is thought to involve inhibition of the LKB1/AMPK signaling pathway. Our study revealed that GPR120, LKB1, and AMPK were under-expressed in PCOS-IR rat ovaries, and LrB treatment upregulated their protein expression levels. LrB may upregulate GPR120 expression and activate the LKB1/AMPK signaling pathway by regulating glucose and lipid metabolism.

NLRP3 inflammasomes are complexes of multiple proteins, including a sensor (NLRP3), an adapter (ASC), and an effector (Caspase-1) [27], which serve as critical mediators of pathological inflammation in many diseases [28]. The NLRP3 inflammasome can be activated by various pathways, triggering Caspase-1-dependent pyroptosis in different cell types [29]. Activation of Caspase-1 can upregulate IL-1β and IL-18 [30]. Studies indicated that the NLRP3 inflammasome contributed to PCOS through regulation of steroid metabolism, n-glycan biosynthesis, post-translational processing, oocyte maturation, autophagy, and apoptotic gene expression [31]. Excessive NLRP3 promotes GC pyroptosis and ovarian fibrosis, leading to the disruption of follicle formation and development and steroid synthesis [32]. Caspase-1 protein expression is significantly greater in PCOS patients than in healthy individuals [33]. Interleukin-1β (IL-1β) is a crucial trigger for the inflammatory response and plays a significant role in proliferation, differentiation, and apoptosis [34]. Others have reported elevated serum IL-1βlevels in both obese and nonobese women with PCOS [35]. IL-18 increases androgen secretion by increasing CYP17A1 and CYP11A1 expression [36], thereby exacerbating PCOS symptoms. TNF-α is an inflammatory cytokine and an important factor that exacerbates insulin resistance and causes dyslipidemia [37]. TNF-α is present in oocytes, granulosa cells, and the corpus luteum and is involved in follicular development, steroidogenesis, ovulation, and corpus luteum function [38]. It is present at relatively high levels in PCOS patients [39]. Interleukin-6 (IL-6) has many functions and is potentially involved in the onset of insulin resistance [40]. Studies indicate higher IL-6 levels in PCOS patients than in healthy women [41]. In our study, LrB was found to reduce NLRP3 inflammasomes and Caspase-1 and lower the serum content of TNF-α and IL-1β, -6, and -18, suggesting that LrB could reduce chronic inflammation in PCOS-IR patients.

LH and testosterone (T) levels in PCOS patients have been reported to be positively correlated with insulin levels [42]. Insulin stimulates the biosynthesis of testosterone in human ovarian thecal cells by activating its own receptor and the inositolglycan mediators’ system. Ovarian granulosa cells are dependent on androgens synthesized by thecal cells as an aromatase substrate for estrogen synthesis. However, insulin significantly increases androgen production in cultured ovarian cells of women with PCOS [43]. In addition, insulin raises androgen levels by increasing the sensitivity of the adrenal cortex to adrenocorticotropic hormone [44]. Excess androgens upregulate anti-mullerian hormone (AMH) levels and inhibit steroid-associated gene expression in granulosa cells of patients with PCOS, thereby inhibiting folliculogenesis and ovulation [45]. Excess androgens may also reduce E2 levels by promoting apoptosis in ovarian granulosa cells [46]. As a result, hyperinsulinemia promotes the development of follicular cysts in rat ovaries [47] and exacerbates the symptoms of PCOS. Elevated LH levels and increased LH/FSH ratios contribute to higher androgen secretion, while reduced FSH levels disrupt the follicular endocrine process [48]. In this study, a PCOS-IR rat model was established using letrozole, an inhibitor that prevents the conversion of androgens to estrogens. Serum LH and T levels were significantly increased, while FSH and E2 were significantly decreased in PCOS-IR rats. After treatment with LrB, LH and T secretions were significantly reduced, and FSH levels were significantly elevated, thus restoring the levels and ratios of these hormones to normal. LrB may regulate sex hormone concentration by lowering insulin levels.

## 4. Materials and Methods

### 4.1. Animals

Ninety female SD rats aged three weeks [49], with an average weight of 70 ± 10 g, were purchased from Hunan Slac Laboratory Animal Co. (Changsha, China). Animal care and handling were performed according to the guidelines, and approval was obtained from the Ethics Committee of the Guangxi University of Chinese Medicine (Approval No. DW20240603-142). The rats were housed in rooms maintained at 20–25 °C with a 12/12 h light cycle and allowed continuous food and water during an adaptation period of one week before this experiment. Ten rats were selected as the normal group, fed a standard diet, and orally gavaged with 0.5% carboxymethyl cellulose sodium each morning at 10 a.m. The other 80 rats received a high-fat diet (HFD) (containing 61.5% ordinary feed, 12% lard, 5% cane sugar, 5% milk powder, 5% peanuts, 10% eggs, 1% sesame oil, 0.5% salt) and orally gavaged with letrozole (1 mg/kg, dissolved in 0.5% carboxymethyl cellulose sodium) each morning at 10 a.m. for 21 days. Vaginal smears were obtained on the 10th day of HFD, and the rats were fed letrozole to screen the PCOS rat models. Rats were fasted from 8 p.m. on the 21st day, and blood was obtained from the tail vein of PCOS model rats at 8 a.m. on the 22nd day to measure fasting blood glucose (FBG) and serum insulin (INS) levels. Insulin resistance (IR) was measured via homeostatic model assessment (HOMA-IR), where the HOMA-IR score = FBG × INS/22.5. Fifty rats with HOMA-IR scores > 2.8 were chosen as the PCOS-IR model for subsequent study [50].

### 4.2. Treatment

**Figure 13:**
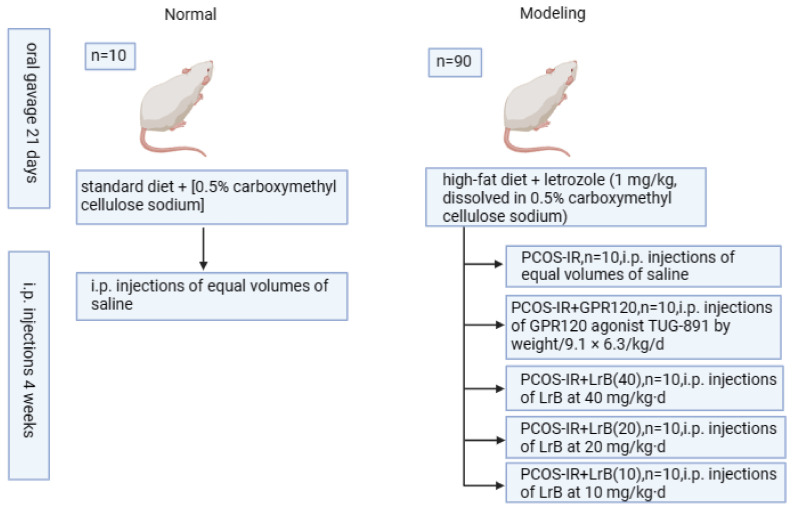
Animal grouping and handling procedures.

Fifty PCOS-IR rats were randomly divided into 5 groups (n = 10/grp): PCOS-IR; PCOS-IR + GPR120 agonist (i.p. injections of GPR120 agonist TUG-891 by weight/9.1 × 6.3/kg·d) [12]; PCOS-IR + high-dose LrB (i.p. injections of LrB at 40 mg/kg·d); PCOS-IR + medium-dose LrB (i.p. injections of LrB at 20 mg/kg·d); PCOS-IR + low-dose LrB (i.p. injections of LrB at 10 mg/kg·d) [17]; and the normal and PCOS-IR groups received i.p. injections of equal volumes of saline. The duration of drug intervention was four weeks, during which food intake did not change for all groups. Modeling, rearing methods, and treatments are shown in Figure 13.

### 4.3. Vaginal Smears

To identify the estrous status of the rats, vaginal smears were collected, air-dried, and stained with hematoxylin and eosin. The estrous cycle stages were observed by visible-light microscopy.

### 4.4. Body Weight (BW) and Lee’s Index Measurements

The BWs of the rats were measured every Monday morning at 10 a.m. At the end of the experiment, the body length (straight line from nose to anus) of each rat was measured, and Lee’s index = [BW (g) × 1000]^1/3^/body length (cm) [51].

### 4.5. FBG Measurement

The day before the end of drug administration, the rats were fasted from 20:00 to 08:00 the following morning, when FBG levels were measured via a glucometer from blood drawn from the tail vein.

### 4.6. Blood and Tissue Sampling

After fasting, blood glucose levels were measured; the rats were i.p.-injected with 3% pentobarbital sodium (0.2 mL/100 g BW), and blood samples were collected from the abdominal aorta. The blood was allowed to clot at room temperature (RT) for 2 h, and the serum was collected by centrifugation at 1006.2× *g* for 20 min at 4 °C for subsequent experiments. Next, ovarian tissue samples were dissected, extraneous fat was removed, and both ovaries were weighed and the values averaged. The long (L) and short (S) diameters were measured, and the ovarian area (A = L × S), ovarian volume (V = 4.19 × [(L + S)/2]^3^) [52], and ovarian index (OI = ovarian weight/rat weight × 10^−3^) [53] were calculated. The left ovaries were stored in liquid nitrogen for subsequent experimental analysis, and the right ovaries were fixed in 4% paraformaldehyde for 24 h and embedded in paraffin.

### 4.7. Hormone Level Measurement

The levels of insulin (INS), follicle-stimulating hormone (FSH), luteinizing hormone (LH), testosterone (T), and estradiol (E2) were detected via enzyme-linked immunosorbent assay (ELISA) kits (Wuhan Cloud-Clone Corp., Wuhan, China), according to instructions. The following specific kits were used for the rats: INS (CEA448Ra); FSH (CEA830Ra); LH (CEA441Mu); T (CEA458Ge); and E2 (CEA461Ge).

### 4.8. Inflammatory Cytokine Assessment

Serum levels of tumor necrosis factor-α (TNF-α), interleukin-1β (IL-1β), interleukin-6 (IL-6), and interleukin-18 (IL-18) were determined by ELISA kit from Jiangsu Enzyme Free Technology Co., Ltd. (Jiangsu Enzyme Free Technology Co., Ltd., Yancheng, China). The following specific kits were used for the rats: TNF-α (MM-0180R1); IL-1β (MM-0047R1); IL-6 (MM-0190R1); and IL-18 (MM-0194R1). All ELISA procedures were performed according to the instructions.

### 4.9. Measurement of Lipid Metabolite Levels

The concentration of total cholesterol (TC), triglycerides (TGs), low-density lipoprotein (LDL), and high-density lipoprotein (HDL) were measured with the following rat-specific ELISA kits following instructions: TC (FT-PD6580S); TG (5878S); LDL (FT-PD5816S); and HDL (FT-PD5888S), all of which were from Jiangsu Enzyme Free Technology Co. (Jiangsu Enzyme Free Technology Co., Ltd., Yancheng, China).

### 4.10. Examination of Ovarian Tissue for Pathology

Paraffin-embedded ovarian tissues were cut into 4 μm sections, deparaffinized in xylene, rehydrated with ethanol, and stained with H&E. The histological characteristics of each sample were observed and analyzed via an Olympus BX51 microscope (Olympus, Tokyo, Japan).

### 4.11. Western Blot

Rat ovarian tissues from liquid nitrogen were thawed at 37 °C and homogenized in RIPA lysis buffer + PMSF (100:1, *v*/*v*) (Solarbio, Beijing, China) at a ratio of 1 mg of tissue to 100 μL of buffer. The protein concentration in the extracts was measured by BCA assay (Solarbio, Beijing, China), and aliquots were run on a 10% SDS—PAGE gel and blotted to a PVDF membrane. The membranes were incubated with 5% nonfat milk in TBST (Tris-buffered saline containing 0.1% Tween-20) for blocking and then overnight at 4 °C with primary antibodies: anti-GPR120 (1:1000, AF5219); anti-NLRP3 (1:1000, DF15549); anti-Caspase-1 (1:1000, AF5418); and anti-glyceraldehyde 3-phosphate dehydrogenase (GAPDH (1:1000, AF7021), all purchased from Affinity (Affinity Biosciences Co., Ltd.,Liyang, China). After washing, the blots were incubated with horseradish peroxidase-conjugated secondary antibodies (1:3000, S0009, Affinity Biosciences Co., Ltd., Liyang, China) at RT for two hours. The proteins were visualized by enhanced chemiluminescence (ECL) (CW0049M, CWBIO) and detected with a ProteinSimple FluorChem E chemiluminescence imaging system (USA). Image-Pro Plus 6.0 software was used to quantitate the grayscale intensity of each protein band, which was normalized to that of GAPDH to calculate the relative expression of each protein.

### 4.12. RNA Purification and Real-Time Quantitative Polymerase Chain Reaction (RT-qPCR)

Total RNA was isolated from ovarian tissues using chloroform and TriQuick reagent (R1100, Solarbio Technology Co., Ltd., Beijing, China), and the concentration and integrity were determined. The RNA was reverse-transcribed to cDNA with the EnzyArtisan cDNA kit (R201-02, EnzyArtisan Biotechnology Co., Ltd., Shanghai, China) in 20 μL reactions: 5 min at 25 °C; 15 min at 37 °C; and 5 s at 82 °C. RT-qPCR amplification was performed using the EnzyArtisan real-time fluorescent quantitative PCR amplification kit (Q204-01, EnzyArtisan, China) with 2 μL of cDNA template in a 20 μL reaction, and specific primers for target genes upstream and downstream were added. PCR amplification was carried out in a Roche LightCycler96 PCR instrument (Roche Diagnostics GmbH, Mannheim, Germarry) under the following conditions: pre-denaturation at 95 °C for 30 s; denaturation at 95 °C for 10 s; and annealing and extension at 60 °C for 30 s, for 40 cycles. The specificity of the PCRs was determined based on amplification curves and melting curves. The relative expression levels of genes in each sample were normalized to those of GAPDH and determined by the 2^−ΔΔCt^ method. The sequences of PCR primers (Wuhan Saier Biotechnology Co., Ltd., Wuhan, China) are provided in Table 1.

### 4.13. Immunohistochemistry to Determine the Expression of LKB1 and AMPK in Rat Ovarian Tissues

Paraffin-embedded rat ovarian tissues were cut into 4 μm sections. The sections were deparaffinized in xylene, hydrated in graded ethanol, and heated for 8 min at 95 °C or epitope retrieval in citrate buffer. After cooling to RT, the sections were treated with H2O2 at RT for 10 min. Subsequently, LKB1 (1:2000, GB111236-100) and AMPK (1:500, GB113685) antibodies from Servicebio (Beijing, China) were added and incubated overnight at 4 °C. The following day, the sections were washed with PBS, incubated with a secondary antibody enhancer at 37 °C for 20 min, washed again with PBS, incubated with a secondary antibody at 37 °C for 20 min, and washed once more with PBS. The sections were developed with DAB for 2–5 min until brown staining appeared, and the reaction was terminated with tap water. The sections were stained with hematoxylin for 2 min, differentiated, rinsed with tap water, dehydrated through graded ethanol, cleared in xylene, mounted, and examined under a microscope. One random field (40× magnification) per rat ovarian section was selected for analysis, and the presence of brown staining was considered positive. The integrated optical density (IOD) values of each field were quantified via Image-Pro Plus 6.0.

### 4.14. Statistical Analysis

The data were processed using SPSS 27.0 and expressed as mean ± standard deviation. Student’s *t*-test was used to compare two groups, and one-way analysis of variance (ANOVA) was used for comparing several groups. When variances were normally distributed, the homogeneous, post-hoc tests were performed via the least significant difference (LSD) method. In cases of inhomogeneous variances, Dunnett’s T3 test was applied. A *p*-value of <0.05 was considered statistically significant.

## 5. Conclusions

Our results confirmed that LrB could upregulate the protein expression of GPR120 in the ovaries of PCOS-IR rats, inducing the LKB1/AMPK signaling pathway and alleviating symptoms in the letrozole-induced PCOS rat model. LrB treatment significantly reduced body weight, ovarian area, and ovarian volume, and the ovarian index in PCOS rats improved the polycystic state of the ovaries, lowered serum LH and T levels, and increased the serum FSH and E2 levels. LrB decreased fasting blood glucose and insulin levels and reduced lipid content in PCOS rats. Furthermore, LrB can reduce inflammation and insulin resistance in individuals with PCOS by inhibiting NLRP3, Caspase-1, TNF-α, IL-6, IL-1β, and IL-18 and increasing the expressions of GPR120, LKB1, and AMPK.

## Figures and Tables

**Table 1:** Primer sequences.

Gene Symbol	Forward Primer	Reverse Primer
GPR120	TTCTTCTCCGATGTCAAGGGC	CGCTTAGGGTCATCACGTAGAAG
NLRP3	GGCCTGTGTGGGAACAAGTA	GGGCGTCCTCTAGTAGTTTCG
CASP1	TGCTTTCTGCTCTTCAACACC	ACCAGGCATATTCTTTCATGTGT
GAPDH	CTGGAGAAACCTGCCAAGTAT	GGTGGAAGAATGGGAGTTGCT

GPR120, free fatty acid receptor 4; NLRP3, NACHT, LRR, and PYD domain-containing protein 3; CASP1, Casparian strip membrane protein 1.

## Data Availability

The datasets used and analyzed during the current study are available from the corresponding author upon reasonable request.
